# Concurrent Performance of Executive Function during Acute Bouts of Exercise in Adults: A Systematic Review

**DOI:** 10.3390/brainsci11101364

**Published:** 2021-10-17

**Authors:** Kefeng Zheng, Liye Zou, Gaoxia Wei, Tao Huang

**Affiliations:** 1 Department of Physical Education, Shanghai Jiao Tong University, Shanghai 200240, China; z757068@sjtu.edu.cn; 2 Exercise Psychophysiology Laboratory, Institute of KEEP Collaborative Innovation, School of Psychology, Shenzhen University, Shenzhen 518060, China; liyezou123@gmail.com; 3 Key Laboratory of Mental Health, Institute of Psychology, Chinese Academy of Sciences, Beijing 100864, China; weigx@psych.ac.cn; 4 Department of Psychology, University of Chinese Academy of Sciences, Beijing 100049, China

**Keywords:** brain, exercise, exercise intensity, executive function

## Abstract

The purpose of the study was to systematically review the evidence on the effects of an acute bout of exercise on concurrent performance of core executive function (EF) during exercise in adults. Four electronic databases (i.e., PubMed, Web of Science, PsycINFO, and SportDiscus) were searched from inception dates to 30 December 2020. The literature searches were conducted using the combinations of two groups of relevant items related to exercise and executive function. Articles were limited to human studies in adults. The search process, study selection, data extraction, and study quality assessments were carried out independently by two researchers. A total of 4899 studies were identified. Twenty-two studies met our inclusion criteria. Of the 42 reported outcomes in the 22 studies, 13 (31%) of the 42 outcomes showed that core EF performance was enhanced during exercise and 14 (33%) found that core EF performance did not differ from control conditions. Fifteen (36%) found that core EF performance was impaired. Notably, improved EF performances tend to be observed during moderate-intensity exercise, whereas impaired EF performances were more likely to be observed at vigorous-high intensity. The review suggests mixed findings regarding the effects of an acute bout of exercise on concurrent performance of core EF. Exercise intensity seems to influence the effects. The underlying neural mechanisms remain to be elucidated.

## 1. Introduction

Accumulating evidence indicates that exercise is not only beneficial to physical health but also to brain and cognitive function [1,2,3], and that the exercise-induced positive effects seem to be significantly larger for executive function (EF) compared to other cognitive subdomains [4,5]. EF refers to a subset of higher-order mental skills, primarily encompassing inhibitory control, working memory, and cognitive flexibility. These three core subdomains have attracted substantial attention from the research community, especially its association with school-based academic performance and future career success [6,7]. Observational studies have shown that higher levels of physical activity or greater aerobic fitness are associated with better EF-related performance among various age groups [8,9,10]. Chronic exercise intervention studies further support that exercise could promote cognitive (and brain) development in children and attenuate the progress of age-related cognitive decline [11,12]. In addition, a growing body of studies have also examined the effects of acute exercise on EF [13,14]. Specifically, the concurrent and subsequent performance of EF were both affected by an acute bout of exercise [15]. Several hypotheses have proposed in the literature to explain the mechanisms underlying the effects of exercise on cognitive performance, such as the cardiorespiratory hypothesis, neurogenesis increase hypothesis, synaptic plasticity increase hypothesis, catecholamines increase hypothesis, and cognitive enrichment hypothesis [16,17].

Individuals often confront a situation wherein cognitive skills and physical activities are simultaneously needed [18], which seems to be simulated by the concurrent measures of cognitive performance during exercise. For example, in sports and military activities, successful performances partly depend on the ability to simultaneously handle physical and cognitive loads [19,20]. To this end, it is of great importance to elucidate how the concurrent performance of EF is modulated during exercise. However, some studies show that EF performances appear to be impaired [21,22,23] or improved [15,24] during an acute bout of exercise, whereas others did not observe any influences [25,26]. Collectively, there is a lack of consensus with regard to the changes in core EF during exercise.

A previous review assessed the effects of acute bouts of exercise on cognitive performance during and after an exercise session [27]. They concluded that submaximal aerobic exercise with a duration up to 60 min facilitated specific aspects of information processing, and that extended exercise negatively influenced information processing and memory function. In a later systematic review with meta-analysis, Lambourne and Tomporowski [28] quantitatively analyzed the effects of acute exercise on cognitive function. They found that cognitive performance declined during the initial 20 min and then facilitated after 20 min of exercise. Similar views have been presented in the review by Chang et al. [29], indicating that the time of cognitive test administration during exercise significantly influenced the outcome, such that effects in the first 20 min of exercise were negligible or negative, and effects after 20 min of exercise were positive. Furthermore, Schmit and Brisswalter [30] proposed a fatigue-based neurocognitive perspective of EF during exercise. They demonstrated that EF performance during relatively long exercise would be dynamic rather than steady and that exercise intensity may not be the most crucial factor to explain EF performance. However, in the aforementioned reviews, the included studies examined performance of a diversity of cognitive skills during exercise. Meanwhile, only a limited number of the included studies investigated the effects of acute exercise on core EF while exercising and highlighted the lack of consensus. Obviously, the inconsistent findings regarding EF performance during exercise warrant further investigations.

Taken together, we identified two gaps in the literature. First, there is no systematic review that has exclusively evaluated the core EF performance during acute bouts of exercise. Second, factors that may moderate the concurrent EF performance during exercise are not clear. With these thoughts in mind, the current study aims to systematically review the evidence on the effects of an acute bout of exercise on concurrent core EF performance during exercise in adults.

## 2. Methods

The systematic review was performed according to the guidelines from the Preferred Reporting Items for Systematic Reviews and Meta-Analyses [31].

### 2.1. Data Sources and Search Strategy

The electronic databases PubMed, Web of Science, SportDiscus, and PsycINFO were searched for relevant articles. Articles were retrieved from inception dates to 30 December 2020. The literature searches were conducted using combinations of two groups of relevant items: (“exercise” OR “aerobic exercise” OR “acute exercise”) AND (“executive function” OR “cognitive control” OR “inhibitory control” OR “working memory” OR “cognitive flexibility”).

### 2.2. Study Selection

Two authors independently performed the literature searches. Upon performing the computerized searches, the article titles and abstracts were reviewed in order to identify potentially relevant articles. All potential and relevant articles were retrieved and reviewed at the full text level. In addition, the bibliographies of the included studies were further screened for missing relevant studies. Any disagreements about the study selection were discussed among the authors until a consensus was reached.

### 2.3. Inclusion/Exclusion Criteria

Studies were included if they: (1) published in peer-reviewed journals with full text available in English; (2) investigated core executive function performance during an acute bout of exercise in adults (18–65 years); and (3) employed an experimental design with a comparison to a no-exercise control group/condition. Studies without predefined exercise intensity (e.g., self-paced cycling or walking) were excluded. Studies conducted in a special condition (e.g., severe hypoxia or breakfast omission) were also excluded.

### 2.4. Methodological Quality of Included Studies

Two authors independently evaluated the methodological quality of the included studies. The methodological quality of each included study was evaluated using the Physiotherapy Evidence Database (PEDro) scale [32,33]. This scale consists of 11 items to assess the methodological quality: eligibility criteria, randomization, concealed allocation, similar baseline, blinding of all subjects, blinding of all therapists, blinding of all assessors, more than 85% retention, intention to treat analysis, between/within group comparison, and point measures and measures of variability. If an item was described absently or unclearly, the article would be given 0 points; if an item was described clearly, the article would be given 1 point. When disagreements of rating between the two reviewers occurred, they discussed and re-evaluated the discrepant results together until reaching a consensus. Total score was calculated, with a higher score indicating better methodological quality. Given that some of the studies employed a within-subject design, these studies were automatically awarded a point for the baseline comparability item. The item for blinded subjects is given 0 points for all studies, since the blinding of participants is not possible in exercise behavioral intervention studies.

### 2.5. Data Extraction of Included Studies

Detailed information of the included studies was extracted, including the first author, methodological quality, participants description, study design, exercise protocol, time of EF test administration and duration, EF task and subdomain(s), and main results. If study findings suggest divergent effects (e.g., shorter RT but impaired RA) of exercise on EF performance, this systematic review retrieved RT as a measure of EF performance.

## 3. Results

### 3.1. Study Selection and Study Characteristics

**Figure 1:**
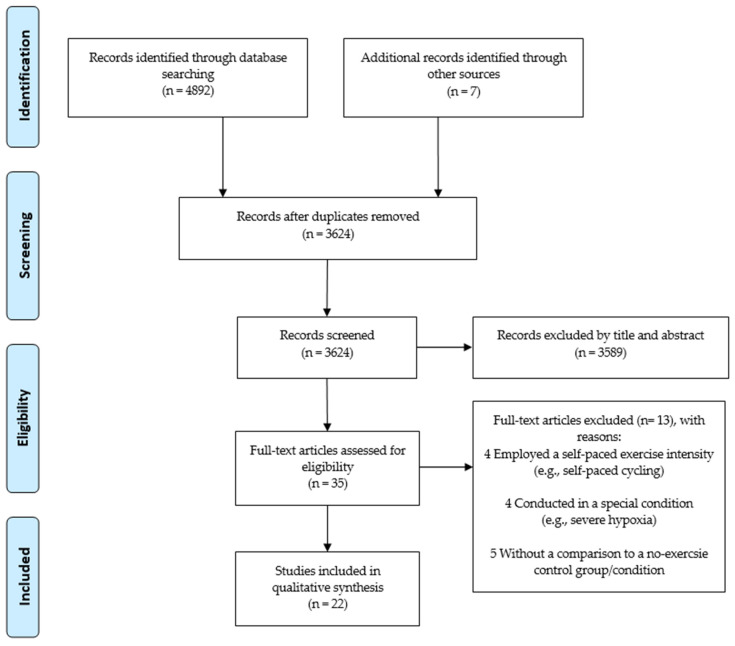
Flow chart of study selection.

Figure 1 depicts the flow chart of the article selection process. The computerized searches identified 4892 articles from the four electronic databases and seven were identified through screening the references in the relevant articles.

After removing duplicates and irrelevant articles, 3624 articles were eligible for further screening. After screening via title and abstract, 35 were identified as potentially relevant, and the full text articles were reviewed. Among them, 13 articles were ineligible as they employed a self-paced exercise protocol (e.g., self-paced cycling), were conducted in a special condition (e.g., severe hypoxia or breakfast omission), or did not employ a comparison to a no-exercise control group/condition. Thus, 22 articles met our inclusion criteria and were included for the qualitative research. Notably, due to the lack of relevant data and the diversity of the experimental protocols (e.g., exercise protocol, time points of EF measurement, and nature of the cognitive tasks), it is not feasible to quantitatively synthesize the findings using a meta-analytic method.

Collectively, in the 22 studies, a total of 590 participants were included, with sample sizes of each individual study ranging from 7 to 120. A total of 13 EF tasks were used. The included studies employed light to high intensities based on classifications of aerobic exercise intensity by Norton et al. [34]. The exercise duration lasted from 7 to 65 min. The study characteristics are summarized in Table 1.

### 3.2. Study Quality

The methodological quality scores are presented in Table 1. The average score of the methodological quality of the 22 studies are 6.5, with scores ranging from 5 to 7 (see Supplementary Table S1 for details).

### 3.3. Study Findings

In the 22 studies, 42 outcomes are reported. Overall, 13 (31%) of these 42 outcomes showed that EF performance was enhanced, whereas 14 (33%) indicated no change in this outcome. Fifteen (36%) found that EF performance was impaired.

Seven of the 42 outcomes, within six studies, employed a light-intensity exercise protocol. Among them, two outcomes demonstrated that EF was improved [24,40], three outcomes found that exercise did not affect EF performance [19,43,47], and another two outcomes found that EF performance was declined [36]. Twenty-four of the 42 outcomes, within 18 studies, employed moderate-intensity exercise protocol. Eight outcomes observed that EF performance was improved [15,24,38,39,42,43]. Eleven outcomes found that EF performance during exercise remained unaltered [19,22,23,25,26,35,39,41,46,47]. Five outcomes found that EF performance was deteriorated [20,22,36,44]. Eleven of the 42 outcomes, within nine studies, employed a vigorous- to high-intensity protocol, of which three found that EF performance was improved [37,40,45]. Eight showed that EF performance was declined [19,21,23,41,46,47]. According to the findings, impaired EF performance most likely occurs during vigorous- to high-intensity exercise. In contrast, the unaltered or even facilitated outcomes of concurrent EF performance are predominantly observed during moderate-intensity exercise. A summary of the outcomes across the varying exercise intensities is presented in Table 2.

The exercise duration of the 22 studies ranged from 7 to 65 min. Six of the twenty-two studies examined the temporal dynamic of EF during relatively long, steady-state exercise (30 min or longer). Two of the six studies found that the improvement of EF performance was greater in the terminal stage compared with earlier stages [42,43]. One study found that the shift towards a less effortful strategy was more pronounced in the first stage of the exercise bout than later [22], which means that inhibitory control was compromised during the initial stage of exercise. Another three studies showed that EF remained unaltered during the entire steady-state exercise session [25,39,45].

Four studies examined EF at several time points (either during or after the first 20 min relative to the onset of exercise). A study by Olson et al. [43] found a significant decline in EF performance either during or after the first 20 min relative to exercise onset. In contrast, one study demonstrated that EF performance was improved after the first 20 min and remained stable during first 20 min relative to the onset of exercise [42]. This is different to the findings of Komiyama et al. [39], who found that EF performance was facilitated both during and after the first 20 min of exercise. In addition, Lambourne et al. [25] revealed that the time points had no significant effect on concurrent EF performance.

Five of the 22 studies investigated cerebral oxygenation during exercise using near-infrared spectroscopy (NIRS). One of the five studies found that EF performance was not associated with changes in cerebral oxygenation sampled at the prefrontal cortex (PFC) [38]. Two showed that EF performance was unchanged or even improved despite a decrease in cerebral oxygenation [40,45]. One study observed that decline in EF performance was related to attenuated cortex oxygenation [19]. Another study revealed that improvement in EF performance was associated with higher cerebral oxygenation, but the change of cerebral oxygenation was not significant [39]. Four studies (two of the four studies also used NIRS) used transcranial Doppler to assess cerebral blood flow (CBF) velocity. They found that improvements or impairments in EF during exercise are not directly related to alterations in CBF [23,38,40,42].

## 4. Discussion

### 4.1. Main Findings

The current study critically reviewed the evidence regarding how the concurrent performance of core EF is affected during an acute exercise session in adults. The available studies revealed mixed findings. Collectively, the findings of the current review indicate that exercise intensity is a potential factor influencing the core EF performance while simultaneously performing exercise. Improved EF performances tend to be observed during moderate-intensity exercise, whereas impaired EF performances during exercise were more likely to be observed at vigorous-to high-intensity.

### 4.2. Role of Exercise Intensity

Although the included studies depicted a picture of mixed findings, exercise intensity seems to moderate the effects of exercise on concurrent EF performance. Most outcomes of these studies found that EF performance during light to moderate intensity exercise remained unchanged or even improved. With regard to the studies that employed a vigorous- to high-intensity protocol, most of outcomes found that EF performance was impaired. Meanwhile, most of the facilitating outcomes were observed during moderate-intensity exercise. The findings are inconsistent with previous studies by Lambourne and Tomporowski [28] and Chang et al. [29], who suggested that exercise intensity might not be a crucial factor influencing the exercise–cognition relationship. The plausibility of our findings can be partly explained by the inverted-U shape relationship between arousal and mental information processing [27,48,49]. Moderate-intensity exercise may lead to a moderate level of arousal [50], thereby facilitating the EF performance during exercise.

According to the reticular-activating hypofrontality model (RAH) proposed by Dietrich and Audiffren [51], it is hypothesized that, within the context of cognitive and physical demands, exercise engages arousal mechanism in the reticular-activating system and disengages the higher-order functions of the prefrontal cortex partly due to the limited resources. As the findings in this review found, the transient hypofrontality most likely occurs during vigorous- to high-intensity exercise. In contrast to the inhibition predicted by the RAH model, these findings suggest that, during moderate-intensity exercise, EF performance can be either unchanged or even facilitated.

### 4.3. Role of Participants’ Physical Fitness and Exercise Mode

In addition to exercise intensity, previous studies suggest that factors such as physical fitness level of participants and exercise mode may moderate the exercise-cognitive function relationship. In the review by Chang et al. [29], physical fitness level was found to be a moderator of the relationship. Positive effects were evident for high-fitness participants, while negative effects were observed for low-fitness counterparts. Unfortunately, the included studies of this review did not examine the potential moderating effects of physical fitness. It is, therefore, not possible to clarify whether the effects of an acute bout of exercise on the performance of core EF during exercise differ among participants with different fitness levels. Most of the included studies employed a cycling protocol. It is also not possible to clarify the moderating effects of exercise mode. However, one included study by Dodwell et al. [37] found that the concurrent performance of working memory was improved during running but not during cycling. Therefore, future studies may further investigate the moderating effects of physical fitness level and exercise mode.

### 4.4. Time Point of the EF Task Administration

Four of the included studies examined the temporal change of EF during exercise and the direction of EF change is mixed [25,39,42,43]. The current findings suggest that the time point of the EF task administration did not significantly moderate the effects of concurrent EF performance. Another two studies measured EF at 20 min following the onset of exercise and found that EF performance was impaired [21,36]. However, these findings are in contrast with a previous review by Lambourne and Tomporowski [28], which suggests that improved cognitive performance occurred when measured after the first 20 min relative to the onset of exercise. Unlike the studies by Lambourne and Tomporowski [28] and Chang et al. [29], which reviewed acute exercise on both higher-order cognitive processes and basic-cognitive processes, the present review focused solely on core EF. Therefore, the inconsistent findings may be driven in part by the nature of cognitive tasks.

This systematic review also sheds light on the importance of considering EF performance as a dynamic process during relatively long and steady-state exercise. For the study conducted by Schmit et al. [37] aimed at investigating inhibitory control during steady-state exercise to exhaustion, EF was measured at an initial and a terminal period of the exercise session. They found that EF was enhanced during the first part of the exercise session, while no sign of deficit was observed shortly before exhaustion. However, individuals’ susceptibility to making errors was increased during the terminal period. Based on those findings, Schmit and Brisswalter [28] proposed a fatigue-based neurocognitive perspective of EF during prolonged, steady-state exercise. From this perspective, EF performance during exercise would be dynamic rather than steady (i.e., positively then negatively impacted by exercise). Apart from that, Olson et al. [43] also examined temporal dynamic of EF during steady-state exercise. EF was assessed at three different time points during the stage of a 31-min cycle. Behavioral findings revealed impaired response accuracy, while faster reaction time was observed at 25 min relative to earlier time points. Although the findings are mixed, it is possible that the performance of EF are dynamic rather than steady during steady-state exercise.

### 4.5. Neural Physiological Responses

In order to elucidate the neural physiological responses, several included studies examined the relationship between CBF or cerebral hemodynamic responses and EF performance during exercise. CBF did not relate to the changes in EF during exercise in all of the included studies [23,38,40,42]. One study using NIRS found that the changes in cerebral hemodynamic responses were not directly related to the changes of EF [38]. However, other studies found a negative or positive link between cerebral hemodynamic responses and EF performance [19,39,40,45]. Overall, the conflicting results from NIRS studies might be due to the different experimental protocols. Moreover, the insufficient spatial resolution of NIRS may partly explain the inconsistent findings.

### 4.6. Strengths and Limitations

The findings of this systematic review have some potential practical implications, since individuals often confront a situation wherein cognitive skills and physical activities are simultaneously needed. It is of importance to understand how the concurrent EF performance is modulated during exercise. Unlike other similar reviews, this study focused solely on concurrent performance of core EF during an acute bout of exercise in adults. To some extent, it eliminates the confounding effects from a diverse cognitive process and helps draw a clearer picture of the concurrent performance of core EF during exercise. However, the review must be interpreted within the context of its potential limitations. First, the included studies employed a diversity of experimental protocols (e.g., exercise protocol, EF test administration and duration, cognitive tasks). The diversity and lack of relevant data make it impossible to perform a meta-analysis. Second, although the outcomes measures consist of three core EFs in this review, the insufficient number of studies limits the possibility of clarifying the potential domain-specific effects on core EF. Lastly, the language of included studies was limited to English, which may lead to the omitting of potential studies published in other languages.

### 4.7. Perspectives and Future Direction

Considering the findings presented in this systematic review, future studies are needed to investigate the concurrent EF performance during exercise in individuals with different fitness levels. It is also interesting to clarify the effects of different exercise modes (e.g., cycling or running) on concurrent EF performance. Furthermore, the possible temporal dynamic of EF at different time points is worth investigating during relatively long exercise. Although the findings tend to suggest that exercise intensity may be a potential factor to moderate the concurrent EF performance during exercise, the underlying mechanisms and neural responses are not clear. Future neuroimaging studies with more rigorous designs are needed to look into the neural responses, thereby elucidating the neural mechanisms of the observed findings.

## 5. Conclusions

The current review suggests mixed findings of the concurrent performance of core EF during exercise in adults. Exercise intensity seems to be a potential factor influencing the effects. The underlying neural mechanisms remain to be elucidated.

## Figures and Tables

**Table 1:** Characteristics of the included studies.

Study (Authors, Methodological Quality)	Participants Description	Study Design	Exercise Protocol	Time of EF Test Administration and Duration	EF Task	EF Domain	Results
Audiffren et al. [22] 5/11	Female: 21.11 ± 1.05 Male: 21.14 ± 0.69 (N = 18) 9M/18	Within-subject design	Cycling at 90% VT (moderate intensity); exercise duration (40 min)	Intermittent assessment (a total of 5 times)	RNG	Working memory; inhibitory control	Inhibitory control was impaired while working memory did not differ from the control conditions. The EF modulation can be interpreted as a change in strategy.
Davranche et al. [35] 7/11	30 ± 8 (N = 14) 11M/14	Within-subject design	Cycling at 50% MAP (moderate intensity); Exercise duration (two periods of 20 min cycling)	Four blocks of task trials were performed during a first 15-min period and another four blocks were performed during a second 15-min period.	Flanker task	Inhibitory control	The task performance was not different from the control condition.
Davranche and McMorris [20] 7/11	32 ± 9 (N = 12) 8M/12	Within-subject design	Cycling at VT intensity (moderate intensity); exercise duration (20 min)	The Simon task began at the end of the 3-min warm-up period and performed within the remaining 17-min period.	Simon task	Inhibitory control	According to the Simon effect, inhibitory control (RT) was impaired.
Del Giorno et al. [36] 7/11	20.2 ± 1.1 (N = 30) 17M/30	Within-subject design	Cycling at 75% VT and VT intensity (light and moderate intensity); exercise duration (30 min)	Cognitive tests began at 20 min following the onset of exercise, lasting for approximately 4 min.	CPT; WCST	Inhibitory control; cognitive flexibility	The performance of two tasks (RA) was impaired during exercise at both light and moderate intensities.
Dietrich and Sparling [21] 7/11							
Exp.1	23.7 ± 9.4 (N = 24) 24M/24	Between-subject design	Cycling or running at 70–80% HR_max_ (vigorous intensity); exercise duration (50 min)	Cognitive tests began after 25 min of exercise, lasting for approximately 10 min.	WCST	Cognitive flexibility	For the WCST, the exercise group made significantly more errors compared to the control group.
Exp.2	25.1 ± 6.3 (N = 8) 8M/8	Within-subject design	Running at vigorous intensity; exercise duration (65 min)	After 25 min of exercise, lasting for approximately 28 min.	PASAT	Working memory	For the PASAT, the exercise condition resulted in significantly more errors than the control condition.
Dodwell et al. [37] 5/11	24.5 ± 2.6 (N = 18) 10M/18	Within-subject design	Cycling or running at 65% HRR intensity (vigorous intensity)	The Retro-cue task began following 5–10 min of warm-up period, included 4 blocks of 96 trials.	Retro-cue task	Working memory	RT was facilitated in the exercise condition compared to the control condition.
Joyce et al. [15] 7/11	23 ± 2 (N = 10) 7M/10	Within-subject design	Cycling at 40% MAP (moderate intensity); exercise duration (30 min)	The Stop-signal task was performed whilst cycling after a 4-min warm-up period and lasted approximately 22 min.	Stop-signal task	Inhibitory control	Inhibitory control was improved during exercise (shorter RT without a change in RA).
Joyce et al. [26] 7/11	23 ± 2 (N = 12) 3M/12	Within-subject design	Cycling at 65% of HR_max_ (moderate intensity); exercise duration (30 min)	The Simon task was performed after 5-min warm-up period and lasted approximately 23 min.	Simon Task	Inhibitory control	According to the Simon effect, inhibitory control was unchanged during exercise.
Komiyama et al. [38] 6/11	21.5 ± 3.5 (N = 13) 13M/13	Within-subject design	Cycling at 50% VO_2max_ (moderate intensity); exercise duration (20 min)	The EF tasks were started after a 5-min warm-up period.	Spatial DR task; Go/No-Go task	Working memory; inhibitory control	The task performance (RT) was improved during exercise without sacrificing RA.
Komiyama et al. [39] 6/11	23.0 ± 2.3 (N = 16) 16M/16	Within-subject design	Cycling at heart rate of 140 beats/min (moderate intensity); exercise duration (30 min)	Intermittent assessment (a total of 2 times)	Spatial DR task; Go/No-Go task	Working memory; inhibitory control	RA was not changed in the Spatial DR task; RT was shorter without sacrificing RA in the Go/No-Go task.
Komiyama et al. [23] 6/11	22.1 ± 1.7 (N = 17) 17M/17	Within-subject design	Cycling at 50%VO_2 peak_ (moderate intensity) for 8 min; thereafter, participants cycled at 80% VO_2 peak_ (vigorous intensity) for an additional 8 min.	Participant performed the EF tasks 3 min after commencing each workload.	Spatial DR task; Go/No-Go task	Working memory; inhibitory control	RA of the tasks was impaired during vigorous-intensity exercise, whereas it was not changed during moderate-intensity exercise; RT was not changed during both intensity exercises.
Lambourne et al. [25] 7/11	21.1 ± 1.7 (N = 19) 8M/19	Within-subject design	Cycling at 90% VT intensity (moderate intensity); exercise duration (40 min)	Intermittent assessment (a total of 5 times)	PASAT	Working memory	RA of the task in the exercise condition did not differ from the control condition.
Lucas et al. [40] 6/11	24 ± 5 (N = 13) 7M/13	Within-subject design	Cycling at 30% followed by 70% of HRR (light and vigorous intensity); exercise duration (two 8-min bouts of cycling)	The Stroop task involved 2 blocks of 20 trials.	Stroop task	Inhibitory control	RT was facilitated during exercise. Vigorous-intensity exercise led to greater improvement compared to light-intensity exercise.
Martins et al. [24] 7/11							
Exp. 1	20.50 ± 0.89 (N = 24) 24M/24	Between-subject design	Cycling at moderate intensity; (short duration)	Four blocks lasting approximately 8 min.	PASAT	Working memory	RA of the task was improved during moderate-intensity exercise.
Exp. 2	19.57 ± 0.83 (N = 120) 55M/120	Mixed Multi-factorial experimental design	Cycling at light and moderate intensity; (short duration)	Two blocks lasting approximately 16 min.	Sternberg task	Working memory	Light and moderate intensity exercise lowered the response latency slopes, resulting in improved working memory.
McMorris et al. [41] 7/11	24.32 ± 7.10 (N = 24) 24M/24	Within-subject design	Cycling at 50% and 80% MAP (moderate and vigorous intensity); exercise duration (15 min or until voluntary exhaustion)	Intermittent assessment (a total of 3 times)	Flanker task	Inhibitory control	Vigorous-intensity exercise impaired RT, but moderate-intensity exercise did not change the task performance.
Ogoh et al. [42] 6/11	20.4 ± 0.6 (N = 7) 7M/7	Within-subject design	Cycling at heart rate of 140 beats/min (moderate intensity); exercise duration (50 min)	Intermittent assessment (a total of 4 times)	Stroop task	Inhibitory control	RT was facilitated during exercise without any loss of performance accuracy.
Olson et al. [43] 7/11	20.4 ± 2.0 (N = 27) 16M/27	Within-subject design	Cycling at 40% and 60% VO_2peak_ (light and moderate intensity); exercise duration (31 min)	Intermittent assessment (a total of 3 times)	Flanker task	Inhibitory control	RA was impaired during both light and moderate intensity exercise, but RT was facilitated during moderate-intensity exercise.
Pontifex and Hillman [44] 7/11	20.2 ± 1.6 (N = 41) 15M/41	Within-subject design	Cycling at 60% of HR_max_ (moderate intensity); exercise duration (approximately 11 min)	The Simon task was performed after 5 min of exercise, lasing for approximately 6.5 min.	Flanker task	Inhibitory control	Exercise did not affect RT but showed a decrease in RA for incongruent trials, resulting in impaired inhibitory control.
Schmit et al. [45] 7/11	22.1 ± 0.6 (N = 15) 10M/15	Within-subject design	Cycling at 85% MAP until exhaustion; exercise duration (approximately 7 min)	Participants performed the Flanker task until exhaustion.	Flanker task	Inhibitory control	RT was facilitated during exercise in the initial stage and remained unaltered in the final stage.
Smith et al. [46] 7/11	28 ± 5 (N = 15) 6M/15	Within-subject design	Running at moderate and high intensity; exercise duration (10 min)	The EF task was performed during the last 2 min of exercise.	Go/No-Go task	Inhibitory control	RT was impaired during high-intensity exercise, whereas it was not changed during moderate-intensity exercise.
Stone et al. [19] 5/11	19.6 ± 2 (N = 13) 8M/13	Within-subject design	Conducted at an exercise intensity in an incremental manner; the average duration was between 20–24 min.	The OWAT test was administered throughout the entirety of the graded exercise test.	OWAT	Cognitive flexibility	RA was not changed at an intensity from 20% to 80% HRR, where it was impaired from 80% to 100% HRR.
Wang et al. [47] 7/11	20.51 ± 1.99 (N = 80) 49M/80	Between-subject design	Cycling at 30%, 50%, and 80% HRR (light, moderate, and vigorous intensity); exercise duration (30 min)	The WCST was performed 6 min after exercise onset.	WCST	Cognitive flexibility	Cognitive flexibility (WCST indices) was impaired in the group of vigorous intensity, whereas it was not changed in groups of light and moderate intensity compared to the control group.

M, male; Exp, experiment; WCST, Wisconsin card sorting task; PASAT, paced auditory serial addition task; RNG, random number generation; CPT, contingent continuous performance task; Spatial DR, spatial delayed response; OWAT: operator workload assessment tool; HRR, heart rate range; MAP, maximal aerobic power; PPO, peak power output; VT, ventilatory threshold; RT, reaction time; RA, response accuracy.

**Table 2:** Summary of the core EF performance across the varying exercise intensities.

Intensity	Facilitation	No Effect	Impairment
Light	● ●	● ●●	● ●
Moderate	●●●●● ●●●	●●●●● ●●●● ●●	●●●● ●
Vigorous-high	●● ●		●●● ●● ●●●

The three exercise intensities included: (1) light-intensity exercise (40 < 55%HR_max_, 20 < 40%HRR, 20 < 40%VO_2max_) vs. control, (2) moderate-intensity exercise (55 < 70%HR_max_, 40 < 60%HRR, 40 < 60%VO_2max_) vs. control, (3) vigorous-high intensity exercise (≥70%HR_max_, ≥60%HRR, ≥60%VO_2max_) vs. control. Green ●: inhibitory control, Yellow ●: working memory, Red ●: cognitive flexibility.

## Data Availability

The data used to support the findings of this study are included within the article.

## Supplementary Materials

The following are available online at https://www.mdpi.com/article/10.3390/brainsci11101364/s1, Table S1: Methodological quality scores.
