# Supplementary material for: Concurrent Performance of Executive Function during Acute Bouts of Exercise in Adults: A Systematic Review
Source: Brain Sci. 2021 Oct 17;11(10):1364. doi: 10.3390/brainsci11101364 (PMC8533767; doi:10.3390/brainsci11101364)
Supplement: Supplementary file 1 [file brainsci-11-01364-s001.zip › brainsci-1345670-supplementary.pdf]

Supplementary Table S1

Study	Item 1	Item 2	Item 3	Item 4	Item 5	Item 6	Item 7	Item 8	Item 9	Item 10	Item 11	Total
Audiffren, et al. [22]	1	1	0	1	0	0	0	0	0	1	1	5
Davranche, et al. [44]	1	1	0	1	0	0	0	1	1	1	1	7
Davranche and McMorris [20]	1	1	0	1	0	0	0	1	1	1	1	7
Del Giorno, et al. [38]	1	1	0	1	0	0	0	1	1	1	1	7
Dietrich and Sparling [21]	1	1	0	1	0	0	0	1	1	1	1	7
Dodwell, et al. [47]	1	1	0	1	0	0	0	0	0	1	1	5
Joyce, et al. [15]	1	1	0	1	0	0	0	1	1	1	1	7
Joyce, et al. [26]	1	1	0	1	0	0	0	1	1	1	1	7
Komiyama, et al. [40]	1	0	0	1	0	0	0	1	1	1	1	6
Komiyama, et al. [41]	1	0	0	1	0	0	0	1	1	1	1	6
Komiyama, et al. [23]	1	0	0	1	0	0	0	1	1	1	1	6
Lambourne, et al. [25]	1	1	0	1	0	0	0	1	1	1	1	7
Lucas, et al. [35]	1	0	0	1	0	0	0	1	1	1	1	6
Martins, et al. [24]	1	1	0	1	0	0	0	1	1	1	1	7
McMorris, et al. [42]	1	1	0	1	0	0	0	1	1	1	1	7
Ogoh, et al. [39]	1	0	0	1	0	0	0	1	1	1	1	6
Olson, et al. [37]	1	1	0	1	0	0	0	1	1	1	1	7
Pontifex and Hillman [45]	1	1	0	1	0	0	0	1	1	1	1	7
Schmit, et al. [46]	1	1	0	1	0	0	0	1	1	1	1	7
Smith, et al. [43]	1	1	0	1	0	0	0	1	1	1	1	7
Stone, et al. [19]	1	1	0	1	0	0	0	0	0	1	1	5
Wang, et al. [36]	1	1	0	1	0	0	0	1	1	1	1	7
**Mean**												**6.5**

Note: Item 1 = eligibility criteria; Item 2 = randomization; Item 3 = concealed allocation; Item 4 = similar baseline; Item 5 = blinding of all subjects; Item 6 = blinding of all therapists; Item 7 = blinding of all assessors; Item 8 = more than 85% retention; Item 9 = intention to treat analysis; Item 10 = between/within group comparison; Item 11 = point measures and measures of variability; "0" = absent or unclear; "1" = clearly described. A higher score indicates better methodological quality.
